# WMR Peptide as Antifungal and Antibiofilm against Albicans and Non-Albicans Candida Species: Shreds of Evidence on the Mechanism of Action

**DOI:** 10.3390/ijms23042151

**Published:** 2022-02-15

**Authors:** Angela Maione, Rosa Bellavita, Elisabetta de Alteriis, Stefania Galdiero, Luisa Albarano, Alessandra La Pietra, Marco Guida, Ermenegilda Parrilli, Caterina D’Angelo, Emilia Galdiero, Annarita Falanga

**Affiliations:** 1 Department of Biology, University of Naples ‘Federico II’, Via Cinthia, 80126 Naples, Italy; angela.maione@unina.it (A.M.); dealteri@unina.it (E.d.A.); luisa.albarano@unina.it (L.A.); ale.lapietra094@gmail.com (A.L.P.); marco.guida@unina.it (M.G.); 2 Department of Pharmacy, School of Medicine, University of Naples ‘Federico II’, Via Domenico Montesano 49, 80131 Naples, Italy; rosa.bellavita@unina.it (R.B.); sgaldier@unina.it (S.G.); 3 Department of Chemical Sciences, University of Naples ‘Federico II’, 80125 Naples, Italy; erparril@unina.it (E.P.); caterina.dangelo@unina.it (C.D.); 4 Department of Agricultural Science, University of Naples ‘Federico II’, Via dell’Università 100, 80055 Portici, Italy

**Keywords:** biofilm, *Candida* species, WMR, *Galleria mellonella*, oxidative damage, CLSM

## Abstract

*Candida* species are the most common fungal pathogens infecting humans and can cause severe illnesses in immunocompromised individuals. The increased resistance of *Candida* to traditional antifungal drugs represents a great challenge in clinical settings. Therefore, novel approaches to overcome antifungal resistance are desired. Here, we investigated the use of an antimicrobial peptide WMR against *Candida albicans* and non-*albicans Candida* species in vitro and in vivo. Results showed a WMR antifungal activity on all *Candida* planktonic cells at concentrations between 25 μM to >50 μM and exhibited activity at sub-MIC concentrations to inhibit biofilm formation and eradicate mature biofilm. Furthermore, in vitro antifungal effects of WMR were confirmed in vivo as demonstrated by a prolonged survival rate of larvae infected by *Candida* species when the peptide was administered before or after infection. Additional experiments to unravel the antifungal mechanism were performed on *C. albicans* and *C. parapsilosis*. The time-killing curves showed their antifungal activity, which was further confirmed by the induced intracellular and mitochondrial reactive oxygen species accumulation; WMR significantly suppressed drug efflux, down-regulating the drug transporter encoding genes *CDR1*. Moreover, the ability of WMR to penetrate within the cells was demonstrated by confocal laser scanning microscopy. These findings provide novel insights for the antifungal mechanism of WMR against *Candida albicans* and non-*albicans*, providing fascinating scenarios for the identification of new potential antifungal targets.

## 1. Introduction

The five most common *Candida* species, *Candida albicans*, *Candida glabrata*, *Candida parapsilosis*, *Candida auris*, and *Candida tropicalis*, with *C. albicans* being the most commonly isolated fungal pathogen in clinical settings, are considered responsible for over 90% of reported cases of invasive candidiasis, in which *Candida* spreads to the blood or other body organs. Nonetheless, the non-*albicans Candida* (NAC) have been increasingly reported globally as emerging multidrug-resistant species [1,2] due to the wide use of prophylactic antifungal treatments, the utilization of invasive medical devices and their biofilm formation capacity.

Recurrent infections caused by *Candida* spp. are difficult to treat because of their ability to form a biofilm, a complex three-dimensional architecture of surface-adhering cells embedded in an extracellular matrix (ECM) providing a protected environment for microbes. Unfortunately, the detailed pathogenic mechanism is still not fully understood [3,4].

The increased resistance to antifungals [5,6] and the ability to develop biofilms on biotic and abiotic surfaces advocate the need for novel therapies. The compounds currently available in the clinics are polyenes (Amphotericin B), azoles and echinocandins, which induce increasing resistance, particularly in hospital opportunistic infections [7], as in the case of *C. auris*, known for its high propensity for developing multidrug resistance which favors nosocomial transmission [8].

Antimicrobial peptides (AMPs) represent promising candidates that could address the fight against planktonic *Candida* cells as well as biofilms [9,10,11,12]. AMPs are 12–50 amino acid long peptides that have been discovered in all forms of life, including bacteria, vertebrates, and invertebrate species [9,10,13,14,15]. Based on their physicochemical properties, they likely have a direct activity on the membrane bilayer [16,17], although sometimes they also have an intracellular target. AMPs are generally cationic and amphipathic; their net positive charge enhances electrostatic interactions with anionic bacterial membranes, while the amphipathic structure leads to membrane insertion, destabilization and disruption. Recently, several AMPs proved to also have an antibiofilm activity [18].

Myxinidin is a marine peptide isolated from the epidermal mucus of hagfish (*Myxine glutinosa* L.), with a considerable antimicrobial activity against a wide range of bacteria and yeast and with low cytotoxicity against human cells [19]. A modified version of the myxinidin sequence (WMR) developed in our laboratory showed higher antimicrobial activity against Gram-positive and Gram-negative bacteria [20,21,22]. WMR comprises the addition of a tryptophan residue at the N-terminus, which is likely involved in WMR strong membrane-disruptive activity and a higher number of positively charged amino acids (i.e., arginines) compared to the native sequence. We previously developed nanofibers functionalized with WMR which were shown to significantly inhibit biofilm formation and eradicate the already formed biofilms of *P. aeruginosa* (Gram-negative bacteria) and *C. albicans*, further supporting the hypothesis that WMR is an interesting AMP to be further developed for its antimicrobial and antibiofilm activities [23,24,25].

The main goal of the present study was to determine in vitro the antifungal and antibiofilm activity of WMR on *C. albicans* and four NAC species, namely *C. glabrata*, *C. parapsilosis*, *C. auris*, and *C. tropicalis.* We evaluated the response to WMR treatment by analyzing the expression of some genes involved in oxidative stress, pump efflux and biofilm formation. Further, we evaluated in vivo the effect of WMR on survival rate and virulence using the *Galleria mellonella*-*Candida* spp. infection model. In addition, we analyzed the synergic activity of WMR with the widely used antifungal drug fluconazole (FLC), which presents a high toxicity and is considered responsible for the emergence of drug-resistant strains.

A deeper investigation on the mechanisms of action of WMR on *C. albicans* and *C. parapsilosis* was performed. *C. parapsilosis* is among the most pathogenic NAC species in clinics [26]. In fact, the increasing incidence of *C. parapsilosis* candidemia is related to long-term hospitalization, affinity to the surface of intravascular devices, prosthetic materials or plastics to form biofilms, and also high glucose and fat environments favoring gastrointestinal colonization and transmission [27,28]. With this in mind, we determined the efflux pump activity with rhodamine 6G assays, following exposure to WMR, to detect the effects of the peptide on drug uptake and efflux, and the intracellular and mitochondrial reactive oxygen species (ROS) accumulation. Confocal laser scanning microscopy (CLSM) was used to examine the architecture of the biofilms developed by *C. parapsilosis* in the presence of the peptide, and to investigate the interactions of WMR with the sessile cells.

## 2. Results

### 2.1. WMR Activity In Vitro and In Vivo on Planktonic Cells and Biofilm

The antifungal and antibiofilm activity of WMR was determined on strains of *C. albicans* and non-*albicans*. Table 1 shows the results obtained. WMR exhibited low antimicrobial activity for *C. albicans*, *C. auris*, and *C. glabrata*, since MIC and MFC values were >50 μM. Instead, in the case of *C. tropicalis* and *C. parapsilosis* it was effective at 25 μM.

The fungicidal effect (MFC) corresponded with MIC only for *C. parapsilosis* and was 50.0 µM for *C. tropicalis*, while for all the others was always greater than 50.0 µM. FLC showed antifungal activity with a MIC value ranging from 16.3 µM to >163 µM for all *Candida* strains tested.

All *Candida* strains were able to form biofilms as shown in Figure S1.

**Figure 1:**
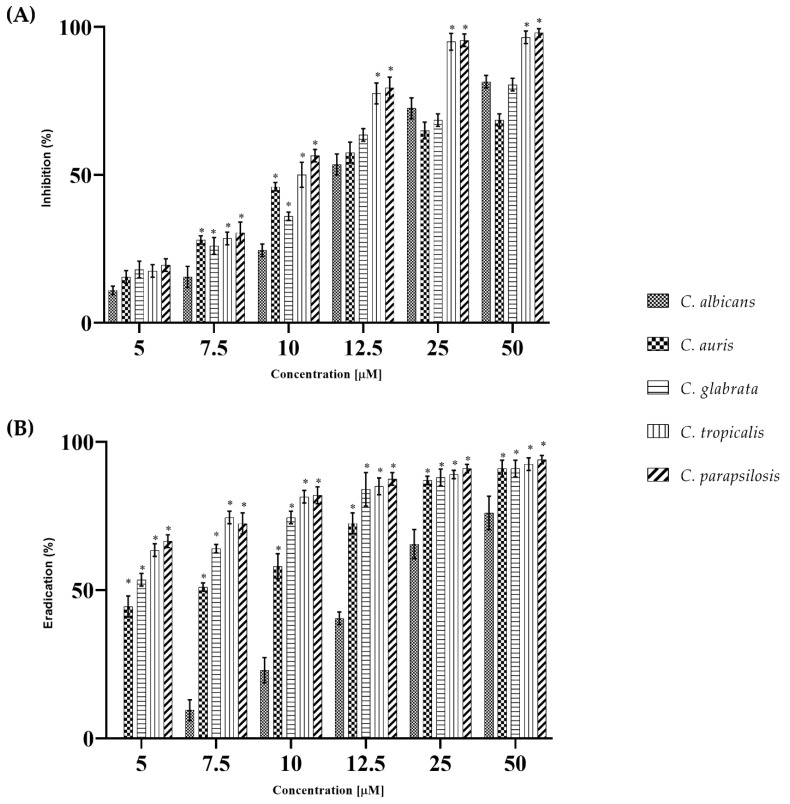
Dose-dependent biofilm inhibition by WMR (5.0; 7.5; 10.0; 12.5; 25.0; 50.0 μM) on *C. albicans, C. auris, C. glabrata, C.tropicalis*, and *C. parapsilosis* after 24 h in triplicate (Panel (**A**)). Dose-dependent biofilm eradication by WMR (5.0; 7.5; 10.0; 12.5; 25.0; 50.0 μM) on *C. albicans*, *C. auris*, *C. glabrata*, *C.tropicalis* and *C. parapsilosis*, in triplicate (Panel (**B**)). * represents significant differences vs. *C. albicans* (Holm–Sidak’s test).

WMR activity was analyzed at increasing concentrations in the two different experimental setups: (1) cells treated with WMR were allowed to form biofilms on the polystyrene surface of microtiter plates (inhibition of the formation); (2) the treatment was done on preformed biofilm (eradication). Although the activity of WMR against planktonic cells was at most moderate, however, the antibiofilm activity was significant. In fact, the development of biofilms was drastically reduced for all *Candida* species (Figure 1 Panel A). In particular, at the highest peptide concentration tested, the best activity was evidenced towards *C. tropicalis* and *C. parapsilosis* with inhibition of almost 100% of biofilm formation, while the biofilm inhibition was around 65% at the same concentration for *C. auris*. The eradication of a mature biofilm is reported in Figure 1 Panel B. Interestingly, in this experiment, the activity of WMR was strong already at the lowest concentrations tested (5.0 µM) with complete eradication of biofilms produced by *C. tropicalis*, *C. parapsilosis* and C. *auris* at the highest concentration tested (50.0 µM).

**Figure 2:**
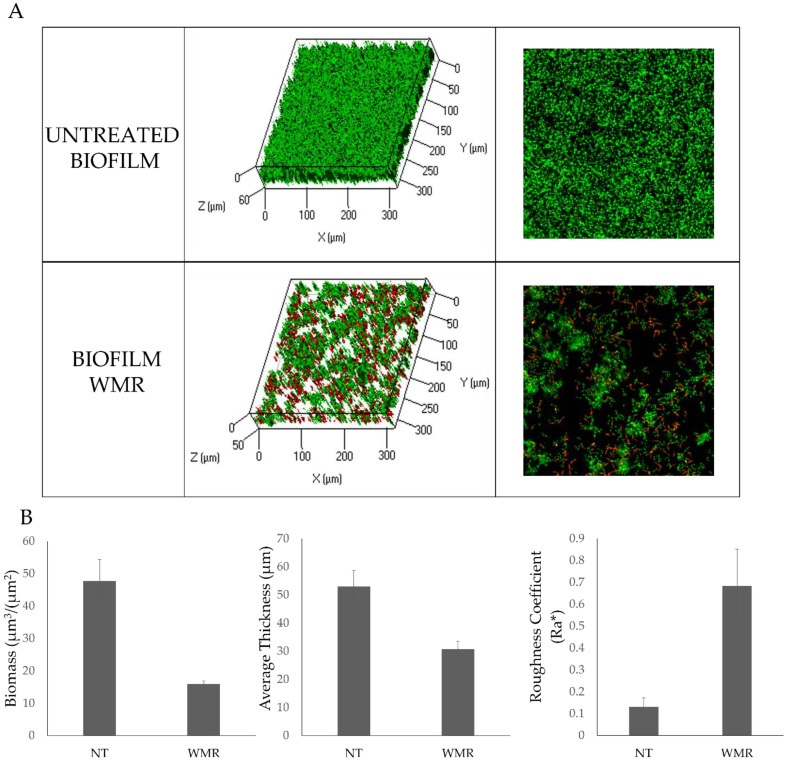
Analysis of the WMR effect on the *C. parapsilosis* biofilm structure. (**A**) CLSM analysis of biofilms formed in the presence (10.0 µM) and absence of WMR. Bi-dimensional and three-dimensional biofilm structures were obtained using the LIVE/DEAD^®^ Biofilm Viability Kit. (**B**) COMSTAT quantitative analysis of biomass, average thickness and roughness coefficient of treated (WMR) and untreated (NT) biofilms.

Biofilm formation of *C.parapsilosis* in the presence of WMR was considered as an explanatory model for the effect of the peptide with respect to biofilm inhibition, since in the case of *C. parapsilosis* 10 μM WMR already gave more than 50% reduction (Figure 1A). Therefore, the antibiofilm activity of WMR peptide on *C. parapsilosis* biofilm formation was also evaluated by confocal laser scanning microscopy (CLSM) (Figure 2). In detail, *C. parapsilosis* biofilm was obtained in the presence of WMR peptide at a concentration of 10.0 µM and then observed by CLSM; Bi-dimensional (SNAP) and three-dimensional (Z-STACK) biofilm structures were obtained using the live/dead staining, indicating viable cells by green fluorescence and red for dead (cell membrane damaged) cells. As shown in Figure 2A, the CLMS analysis demonstrated the WMR capability to reduce *C. parapsilosis* biofilm formation. Moreover, it is interesting to note the higher number of damaged cells in the biofilm formed in presence of WMR, compared to that formed in the absence of peptide (Figure 2A). To get more detailed information about the biofilm structure, the collected three-dimensional images were analyzed using the COMSTAT image analysis software package [29]. Untreated biofilms appeared thick and showed a more compact structure, compared to those formed in the presence of WMR, which appeared less homogeneous and thinner (Figure 2B).

Further insight into antibiofilm potential of WMR was investigated at the gene level by qRT-PCR. Two major transcriptional genes that govern biofilm formation *(ERG11, ALS3/5*)*, HOG1* that is activated in *Candida* strains in response to diverse stimuli and *CDR1* encoding efflux pumps were detected.

**Figure 3:**
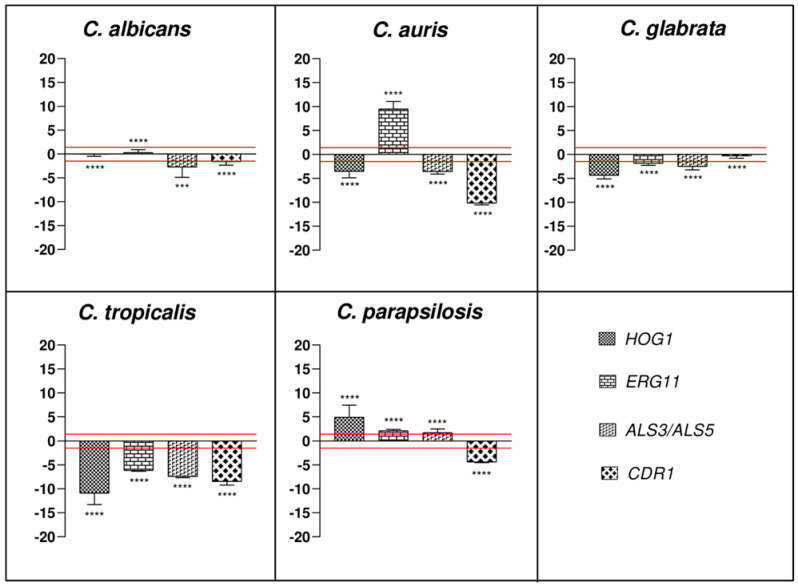
Real-time qPCR. Histograms show the differences in the expression levels of four selected genes, involved in biofilm and structure formation, efflux pumps and in stress response upon exposure to sub-inhibitory concentration of WMR. Fold differences greater than ±1.5 (see red dotted horizontal guidelines at values of +1.5 and −1.5) were considered significant (see Table S2 for the values). Tukey’s test (*** *p* < 0.001; **** *p* < 0.0001).

The relative expressions of *ALS3* (*ALS5* for *C. auris*), *ERG11*, *CDR1* and *HOG1* genes were evaluated for all the five *Candida* during the inhibition of biofilms formation. *HOG1* was targeted by WMR in all *Candida* species with exception of *C. albicans*. Specifically, it was down-regulated in *C. auris*, *C. glabrata* and *C. tropicalis*, and up-regulated in *C. parapsilosis* compared to the control (untreated biofilms). The down-regulation in *C. auris* was statistically significant compared to the expression changes in *C. tropicalis* (*p* < 0.0001), *C. parapsilosis* (*p* < 0.0001) and *C. albicans* (*p* < 0.01), which in turn was statistically significant compared to *C. glabrata*, *C. tropicalis* and *C. parapsilosis* (*p* < 0.0001). Moreover, the expression levels showed in *C. glabrata* were statistically significant compared to those described for *C. tropicalis* and *C. parapsilosis* (*p* < 0.0001), which in turn were statistically significant to each other (*p* < 0.0001; see Figure 3).

Furthermore, *ERG11* was targeted by WMR in all *Candida* species with exception of *C. albicans*, showing a down-regulation in *C. glabrata* and *C. tropicalis* and an up-regulation in *C. auris* and *C. parapsilosis* (Figure 3). The expression level of this gene in *C. auris* was statistically significant compared to the expression changes in *C. tropicalis* (*p* < 0.0001), *C. parapsilosis* (*p* < 0.0001)*, C. albicans* (*p* < 0.0001) and *C. glabrata*, which in turn was statistically significant compared to *C. tropicalis* and *C. parapsilosis* (*p* < 0.01). Moreover, the expression levels showed in *C. tropicalis* were statistically significant with respect to those described for *C. albicans* and *C. parapsilosis* (*p* < 0.0001).

In contrast, *ALS3* (*ALS5* for *C. auris*) gene was targeted in all *Candida species* where there was a down-regulation except for *C. parapsilosis* (see Figure 3). Its expression level change in *C. auris* was statistically significant with respect to the expression changes in *C. tropicalis* (*p* < 0.01) and *C. parapsilosis* (*p* < 0.0001)*,* which in turn was statistically significant compared to *C. glabrata* (*p* < 0.01), *C. tropicalis* (*p* < 0.0001) and *C. albicans* (*p* < 0.001). Moreover, the expression levels showed in *C. tropicalis* were statistically significant with respect to those described for *C. albicans* and *C. glabrata* (*p* < 0.01).

Finally, *CDR1* showed a down-regulation in all *Candida sp* with exception of *C. glabrata*. The down-regulation in *C. albicans* was statistically significant compared to the expression changes in *C. tropicalis* (*p* < 0.0001) and *C. auris* (*p* < 0.0001), which in turn was statistically significant compared to *C. glabrata*, *C. tropicalis* and *C. parapsilosis* (*p* < 0.0001). Moreover, the expression levels showed in *C. tropicalis* were statistically significant compared to those described for *C. glabrata* (*p* < 0.0001) and *C. parapsilosis* (*p* < 0.05), which in turn were statistically significant compared to each other (*p* < 0.01; see Figure 3).

**Figure 4:**
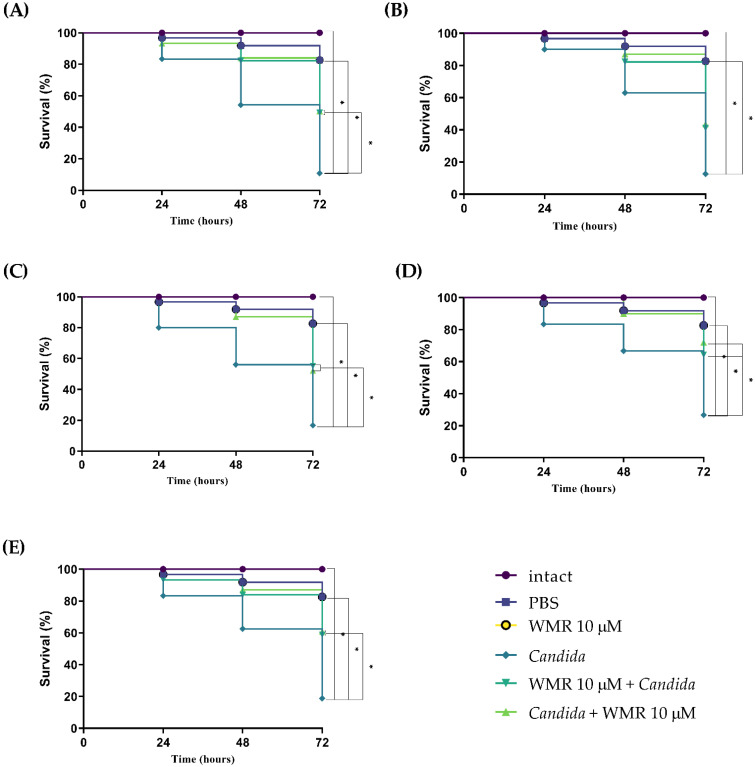
Kaplan–Meier plots of survival curves of *G. mellonella* larvae infected with *C. albicans* (**A**), *C. auris* (**B**), *C. glabrata* (**C**), *C. tropicalis* (**D**), *C. parapsilosis* (**E**). The concentration of *Candida* cells was 1 × 10^6^ CFU/larva. Treatments consisted of phosphate buffered saline (Control), WMR alone (10.0 μM), WMR (10.0 μM) before or after infection; intact larvae (control). The data are the means of three independent experiments. * *p* < 0.05 (Dunnett’s test).

In vivo antimicrobial activity of WMR was evaluated using *G. mellonella* larvae infected with strains as shown in Figure 4. The concentration of 10^6^ cells/larvae caused about 70% larval survival within 24 h after infection and was used in subsequent assays. Survival was 100% in the intact control group and no statistically significant difference was found with PBS control group. The effect of 10.0 µM WMR on larval viability demonstrated the absence of toxicity. Figure 4 shows that the survival of the larvae for all strains tested was around 40–50% for infection with the pathogen alone at 48 h and it increased up to about 80% when larvae were pre- or post-treated with 10.0 µM of the peptide (*p* < 0.05). Even more evident was the increase of larvae survival at 72 h (about 30–40%) in the case of the pre- or post- treatment compared to untreated larvae (*p* < 0.05). No differences were observed for the pre- and post-treatment.

### 2.2. WMR Mechanism of Activity on Planktonic Cells and Localization of FITC-Labeled WMR

**Figure 5:**
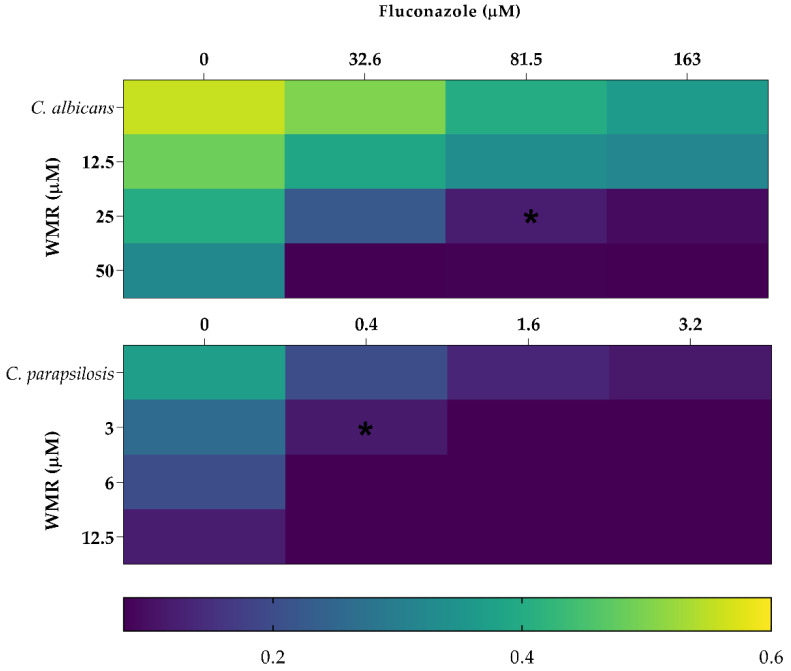
The combined activity of WMR and FLC against planktonic growth of *C. albicans* and *C. parapsilosis*. The color bar indicates relative OD growth. * indicates synergy (FICI ≤ 0.5).

To explore the potential mechanism of WMR we performed further experiments only on two species, namely *C. albicans* and *C. parapsilosis.* They were chosen because *C. albicans* is the reference species among the genus *Candida,* and *C. parapsilosis* was the most susceptible to WMR among the NAC species examined both in planktonic and sessile forms. The growth of *C. albicans* and *C. parapsilosis* was monitored for 24 h using kill curves (Figure S2). The results showed that WMR has fungistatic activity at concentration over 50.0 µM and no fungicidal effect for *C albicans* whereas for *C. parapsilosis* a low dose was needed to detect fungistatic and fungicidal activities (10.0 and 25.0 µM, respectively). The synergic effect of WMR with FLC was analyzed using the checkerboard microdilution assay. We explored the antifungal activity levels of WMR alone and in combination with FLC on both *C. albicans* and *C. parapsilosis* (Figure 5). The best combinations of FLC/WMR were 81.5/25 μM and 3.1/0.4 μM, for *C. albicans* and *C. parapsilosis*, respectively. In both cases a synergistic effect of the two molecules was ascertained since the fractional inhibitory concentration index (FICI) was <0.5. These results clearly indicate that WMR is able to support the antifungal effect of FLC leading to a significant decrease in the MIC values of the two compounds.

**Figure 6:**
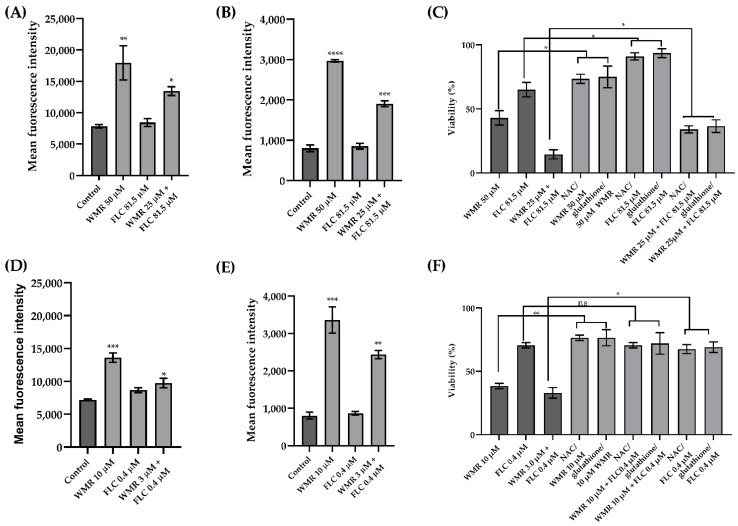
Induction of intracellular (**A**,**D**) and mitochondrial (**B**,**E**) ROS and cell rescue assay using ROS scavengers (**C**,**F**) on *C. albicans* (**A**–**C**) and *C. parapsilosis* (**D**–**F**). The viability was determined by counting CFUs after incubation with the agents and expressed as the percentage of survivals. ns = not significant, * = *p* < 0.05, ** = *p* < 0.01, *** = *p* < 0.001, **** = *p* < 0.0001 (Holm–Sidak’s test). FLC = fluconazole, NAC = N-acetyl cysteine.

A lot of compounds with fungicidal activity induce a common oxidative-damage cellular death pathway, suggesting that at least part of the fungicidal effect depends on ROS production. Here, the intracellular ROS levels in *C. albicans* and *C. parapsilosis* were measured to verify whether they were generated following exposure to WMR, FLC, and to the combinations WMR/FLC which had shown the synergistic effect. The H2DCFDA-positive cells after WMR and WMR/FLC treatment significantly increased compared to untreated cells. Instead, when the cells were treated with FLC alone, ROS level did not increase significantly (Figure 6). Similarly, an increase in mitochondrial O^2−^ was observed after WMR and WMR/FLC treatment. These results confirmed that mitochondria played an important role in the production of ROS.

Subsequently, we examined the effects of ROS production on *C. albicans* and *C. parapsilosis* cell death using NAC and glutathione as a ROS scavenger. NAC treatment increased the survival of the cells of about 40% when treated with WMR (Figure 6). These results demonstrated increased cell survival by NAC pre-treatment compared to WMR treatment alone.

**Figure 7:**
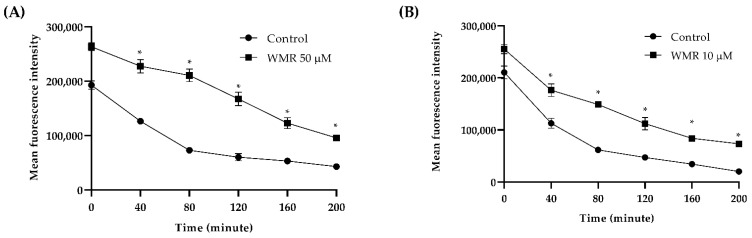
The effect of WMR on the efflux of Rh6 G in *C. albicans* (**A**) and *C. parapsilosis* (**B**). Mean fluorescence intensity represented the intracellular Rh6 G in fungal cells. Statistical significance was determined by Student’s *t*-test. * *p* < 0.05 when compared with the respective controls.

The effect of WMR on the efflux pump activity is shown in Figure 7, where WMR clearly inhibits the activity of the efflux pump at a concentration of 10.0 μM. The fluorescence intensity was greater in treated cells compared to untreated, showing an accumulation of Rh6G and thus indicating inhibition of efflux pump activity.

**Figure 8:**
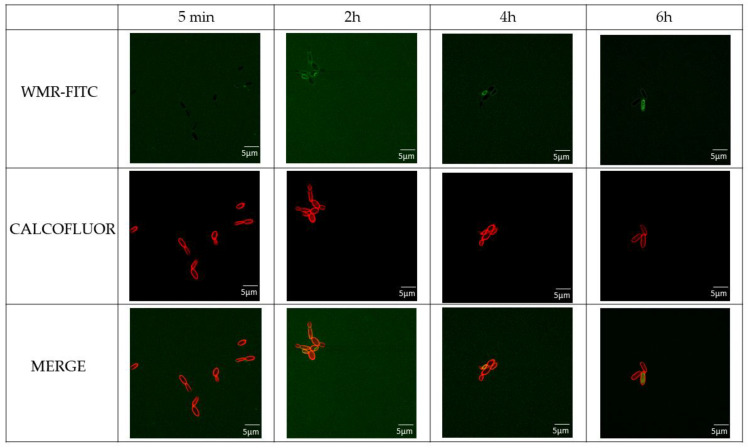
Localization of WMR-FITC peptides. Fluorescence confocal microscopy images of *C. parapsilosis* treated with FITC-labeled WMR peptides (10.0 µM) at different incubation times and stained with Calcofluor white dye. Green fluorescence indicates the peptide and red fluorescence shows the cell wall stained with calcofluor white.

To investigate the molecular mechanism involved in WMR activity, the localization of the peptide was investigated by CLSM. *C. parapsilosis* cells were treated with WMR-FITC at different times and co-stained with Calcofluor white stain marking the bacterial wall with red colour (Figure 8).

FITC-labelled WMR showed slow penetration through *C. parapsilosis* membranes for the first incubation times and accumulated in the bacterial cytoplasm after already 4 h of incubation. Moreover, the treatment with WMR peptide showed an alteration of the morphology of the *C. parapsilosis* cells (Figure S3).

## 3. Discussion

The epidemics related to yeast infections and resistance issues to available antifungal drugs are rapidly increasing; moreover, non-albicans *Candida* species and rare yeast species are emerging as major opportunistic pathogens. We previously developed a peptide WMR with antibacterial and antifungal activity, which was also shown to be effective against *K. pneumoniae* and *C. albicans* biofilms as well as against mixed biofilms [30]. Here, we further investigated the activity of WMR against *Candida* species to unravel the mechanism of action.

As biofilm formation is critical for the development of fungal resistance, we first showed that WMR exhibited antifungal activity against planktonic cells and biofilm cells of *C. albicans* and non-*albicans Candida* species in vitro. In vitro studies are clearly not sufficient to demonstrate the safety and efficacy of drugs; we thus made in vivo experiments mimicking infection in *G. mellonella*, an insect model with obvious advantages in ethics, logistics and economy, that is widely used to quickly assess the efficacy and toxicity of drugs in vivo. Our study demonstrated not only the non-toxicity of the peptide but also a significantly increased survival rate of treated larvae compared to untreated. Interestingly, qRT-PCR studies showed that the expressions of several stress- and biofilm-related genes were simultaneously altered in all *Candida* strains tested.

The results confirmed that the inhibitory effects of WMR in *C. albicans* and *non-albicans* biofilm formation is related to the suppression of major gene expression. Previous studies demonstrated that the inhibition of genes associated with cellular stress and biofilm formation, such as *HOG1, ALS3-5, ERG11,* and *CDR1*, limits cell adhesion, elongation, and pump efflux [31].

Particularly, *HOG1* is activated and promotes resistance to diverse stress conditions likely to be encountered in the host or during antimicrobial therapy. It is known that *HOG1* is important for cellular responses to antimicrobial peptides and particularly is implicated in a multitude of cellular processes in *Candida* strains, such as the yeast to hyphal switch or chlamydospore production, and so is important for virulence [32]. In our study, *HOG1* was significantly upregulated in *C. parapsilosis* showing a correlation with the increase of intracellular ROS and the beginning of an effective oxidative stress response regulated by this transcription factor in order to adapt cells to oxidative stress and above all highlighting a cellular response to osmotic stress.

The upregulation of *ERG11*, involved in the ergosterol pathway, explained the resistance to WMR of the two species *C. auris* and *C. parapsilosis* while it was significantly downregulated in *C. tropicalis* and *C. glabrata*.

WMR treatment significantly diminished the expression of the adhesin gene ALS3/5 encoding for hyphal growth in all strains, which was directly corroborated with the decreased biofilm formation in vitro and reduced colonization in the in vivo *G. mellonella* infection model.

The CLSM analysis of the biofilm confirmed that WMR inhibited the biofilm formation. The analysis of the structural parameters of the biofilm clearly showed a reduction of the biomass and thickness, together with an increase of roughness, which is correlated with the lower homogeneity of the remaining biofilm. The CLSM analysis on the peptide interaction with *C. parapsilosis* planktonic cells demonstrated that peptide action is mainly divided in two steps: first it binds *C. parapsilosis* membranes, then it penetrates in the bacterial cytoplasm where it accumulates; after 6 h of incubation the peptide is mainly present in the cell cytoplasm. It is interesting to note that treatment with WMR peptide resulted in an alteration of the morphology of the *C. parapsilosis* cells. The penetration ability of the peptide explains how the WMR can modulate the expression of genes related to the hyphal growth and other genes involved in the formation of the biofilm. We may hypothesize that AMPs activity is strongly correlated both to their structural features with their high positive charges directly involved in the disruption of the biofilm structure and also to the regulation of several genes involved in the construction and maintaining of the biofilm structure.

In addition, downregulation of *CDR1*, a multidrug transporter gene, signified that WMR-treated fungal cells are less likely to develop antibiotic resistance.

To strengthen the understanding of the peptide activity, other studies were performed to determine the multiple modes of action of WMR on *C. parapsilosis* due to the increased importance of this species to healthcare, compared to *C. albicans*.

Under pathological conditions, the accumulation of intracellular ROS exceeds the metabolic capacity of reductases, eventually triggering cell apoptosis. In this study, WMR induced ROS generation, both total and mitochondrial, that may cause oxidative stress, likely associated with the killing activity of it and corroborating the hypothesis that at least part of the antimicrobial activity of the peptide is due to ROS accumulation. Furthermore, the non-functioning of the efflux pump, evidenced by a greater accumulation of rhodamine, revealed that the higher intracellular Rh6G accumulation in the treatment group implied that more WMR remained in the cell.

In conclusion, WMR exhibited antifungal activity against both planktonic *C. albicans* and non-*albicans* and their biofilms. Synergism was observed when WMR was combined with FLC against *C. albicans* and *C. parapsilosis* strains. Time- killing curves confirmed antifungal effects of WMR in dynamic that were further corroborated in vivo with *G. mellonella* infection model. Mechanism studies showed that WMR could induce both intracellular ROS accumulation and mitochondrial dysfunction. Besides, WMR could promote suppression of drug efflux by down-regulating drug transporters genes *CDR1*.

The synergy of activity between WMR and FLC opens the way to new strategies to address biofilm treatment in clinics. The significant increase in antifungal activity of FLC and WMR when used in combination represents certainly the most interesting result. It is likely that WMR aids in the disruption of the biofilm structure and favors the activity of FLC which could represent an interesting strategy to reduce the dose of the drug and reduce the emergence of resistance issues. As already mentioned, the mode of action of FLC and WMR are extremely different. The major mechanism of action of WMR as of many other AMPs is initially directed towards the biofilm structure, producing a local disruption, which probably facilitates the entrance of conventional drugs such as FLC, promoting a synergistic activity that likely affects targets inside the cells. In conclusion, this study is the first to elucidate the antifungal activity of WMR both in vitro and in vivo and to explore its potential mechanism of activity. These findings might provide insights into the possible therapeutic application of WMR as an antifungal agent or as an enhancer of traditional antifungal drugs.

## 4. Materials and Methods

### 4.1. Candida Strains, Media and Growth Conditions

Five *Candida* strains were used in this study including *Candida albicans* ATCC 90028, *Candida auris* DSM 21092, *Candida glabrata* DSM 11226, *Candida tropicalis* DSM11951 and *Candida parapsilosis* DSM 4874. They were grown on YPD Agar (1% *w/v* yeast extract, 2% *w/v* peptone, 2% *w/v* glucose, 1.5% Agar) and cultured in Tryptone soy broth supplemented with 0.1% glucose for 16–18 h at 37 °C. For subsequent experiments, they were washed twice using sterile phosphate-buffered saline (PBS) and standardized to 10^6^ cells mL^−1^. RPMI 1640 medium (Thermo Fisher Scientific, Waltham, MA, USA) buffered to a pH of 7.0 with 0.165 M MOPS was used for growing the biofilms of all *Candida* species.

### 4.2. Peptide Synthesis

WMR (NH_2_-WGIRRILKYGKRSK-CONH_2_) was synthesized using the ultrasound-assisted solid-phase peptide strategy (US-SPPS) combined with the orthogonal Fmoc/*t*Bu chemistry [33]. Briefly, the peptide (50 μmol) was assembled on the Rink amide MBHA resin (0.54 mmol/g) by consecutive deprotection and coupling cycles; Fmoc-deprotection: 10% piperidine in DMF, 0.5 + 1 min; coupling condition: Fmoc-amino acid (3 equiv), HOBt/HBTU as additive reagents (3 equiv), and DIPEA (6 equiv) as basis, 2 × 10 min. After the elongation of the peptide, the N-terminal Fmoc group was removed and the peptide was cleaved from the resin and its protecting groups by treatment with TFA:TIS:H_2_O (95:2.5:2.5, *v/v/v*) at rt for 6 h. Then, the resin was removed by filtration and the crude peptide was recovered by precipitation with cold ethylic ether. Analysis of the crude peptide was performed by ESI LC–MS using a gradient of acetonitrile (0.1% TFA) in water (0.1% TFA) from 5 to 70% in 15 min. The peptide was purified by preparative RP-HPLC using a gradient of acetonitrile (0.1% TFA) in water (0.1% TFA) from 5 to 70% in 15 min. The purified peptide was obtained with good yields (80%).

### 4.3. Fluorescein-Labelling of WMR

The fluorescein labelling of peptide WMR [sequence: WGIRRILKYGKRSK-K(FITC)] was performed by adding Fmoc-Lys(Mtt) residue at *C*-terminal. The Mtt protecting group was removed in orthogonal condition from lysine with respect to the Fmoc/*t*Bu. The synthesis of the entire sequence was performed by US-SPPS methodology as described above. After the assembly of the peptide sequence, the Mtt group was deprotected treating the resin with a cocktail of TFA:TIS:DCM (1:5:94, *v/v/v*), 7 × 25 min, at rt. Then, the resin was filtered and washed with DMF (×3) and DCM (×3) and the Mtt removal was ascertained by Kaiser test. At this stage, the fluorescein isothiocyanate (6 equiv) was added in presence of DIPEA (12 equiv), at rt, overnight [34]. Then, the resin was washed with DMF (×3) and DCM (×3), N-terminal Fmoc group was removed, and the peptide was cleaved by treatment with TFA:TIS:H_2_O (95:2.5:2.5, *v/v/v*), at rt for 6 h. The peptide was purified by preparative RP-HPLC using a gradient of acetonitrile (0.1% TFA) in water (0.1% TFA) from 5 to 70% in 15 min. The purified peptide was obtained with good yields (70%).

### 4.4. Determination of Minimum Inhibitory Concentrations and Minimum Fungicidal Concentration of Planktonic Cells

The minimum inhibitory concentrations (MICs) of WMR and fluconazole (FLC) against the five *Candida* strains were determined with a broth microdilution method as described by CLSI-M27-A3. [35] Briefly, 50 μL of FLC (0.1–163.0 μM) and WMR (2.0−50.0 μM) were serially diluted in RPMI 1640 medium buffered with MOPS added to the 96-well plate together with the organism suspensions adjusted to an inoculum of 10^6^ cell mL^−1^ and incubated at 37 °C for 24 h. MICs values were determined as the lowest concentration inhibiting fungal growth at 590 nm using a microplate reader (Synergy™ H4; BioTek Instruments, Inc., Winooski, VT, USA). Minimum fungicidal concentration (MFC) values were defined as the lowest concentration that showed no colony growth on the culture medium and were determined by subculturing 10 μL of the medium collected from the wells showing no microscopic growth on YPD after 24 h. The MFC was the lowest concentration that yielded no colonies growth on agar.

### 4.5. Biofilm Formation, Inhibition and Eradication

To develop *C. albicans, C. auris*, *C. tropicalis*, *C. parapsilosis* and *C. glabrata* biofilms, cell suspensions (10^6^ cell mL^−1^) prepared in RPMI 1640 were placed in 96-well polystyrene microtiter plates (100 µL per well), which were incubated for 24 h at 37 °C. After incubation, the medium was removed and the biofilms were washed with 200 µL of phosphate-buffered saline (PBS; 0.01 M) to remove non-adherent cells. The biofilms were quantified using the crystal violet (CV) staining methodology and absorbance was quantified at 570 nm using a microtiter plate reader as described previously [36,37].

To evaluate the biofilm inhibition performance of WMR on these strains, it was added to 96-well polystyrene microplates together at a concentration ranging from 5 μM to 50 μM then incubated for 24 h at 37 °C. To investigate the impact of WMR on mature biofilms (eradication activity), the cells were allowed to adhere to the plates and then incubated with the peptide at the same concentrations for another 24 h. Biofilms vital biomass was quantified by using the tetrazolium 2,3-bis (2-methoxy-4-nitro-5 sulfophenyl)-5-[(phenylamine) carbonyl]- 2H-hydroxide re-duction assay (XTT) (Sigma-Aldrich, St. Louis, MO, USA) according to the manufacturer’s instructions and the absorbance was measured spectrophotometrically at 492 nm. The percentages of inhibition or eradication were calculated as: % biofilm reduction = Abs control − Abs sample/Abs control × 100 [38].

### 4.6. Effect of WMR on Gene Expression during Biofilm Inhibition

To elucidate the potential mechanisms by which WMR inhibits *Candida* biofilms, gene expression analysis of *CDR1*, *ALS3*, *ERG11*, *HOG1* was performed using qRT-PCR. Biofilms were developed with and without 10.0 μM WMR for 24 h. PBS-washed biofilms were then scraped, collected, centrifuged and the pellet was used to extract total RNA using Direct-zolTM RNA Miniprep Plus Kit (ZYMO RESEARCH). The purity and concentration of the extracted RNA were verified using Nanodrop spectrophotometer 2000 (Thermo Scientific Inc., Waltham, MA USA), the concentration by the absorbance at 260 nm and the purity by 260/280 and 260/230 nm ratios [39]. For each sample, 1000 ng of total RNA was retrotranscribed with an iScript™ cDNA Synthesis kit (Bio-Rad, Milan, Italy), following the manufacturer’s instructions.

The variations in expression of genes involved in biofilm and structure formation (*ALS3*/*ALS5* for *C. auris*) and *ERG11*, respectively), efflux pumps operation (*CDR1*), stress response (*HOG1*) and normalizer *ACT1* (see Table S1) were evaluated. Undiluted cDNA was used as a template in a reaction containing a final concentration of 0.3 mM for each primer and 1× SensiFASTTM SYBR Green master mix (total volume of 10 µL) (Meridiana Bioline). PCR amplifications were performed in an AriaMx Real-Time PCR instrument (Agilent Technologies, Inc.Milan Italy), according to the manufacturer’s instructions. The system thermal cycler used the following thermal profile: 95 °C for 10 min, one cycle for cDNA denaturation; 95 °C for 15 s and 60 °C for 1 min, 40 cycles for amplification; 95 °C for 15 s, one cycle for final elongation; one cycle for melting curve analysis (from 60 °C to 95 °C) to verify the presence of a single product. Each assay included a no-template control for each primer pair. To capture intra-assay variability, all real-time qPCR reactions were carried out in triplicate. Fluorescence was measured using Agilent Aria 1.7 software (Agilent Technologies, Inc.). The expression of each gene was analyzed and normalized against the *ACT1* gene using REST software (Relative Expression Software Tool, Weihenstephan, Germany, version 1.9.12) based on the Pfaffl method [40,41]. Relative expression ratios greater than ± 1.5 were considered significant.

### 4.7. Determination of In Vivo Antifungal Effects Using the G. mellonella Infection Model

*G. mellonella* larvae were selected to be absent of dark spots and similar in size (approximately 250–300 mg each). Each group containing 20 randomly chosen larvae was used for every treatment as previously reported [42]. Larvae were cleaned by an alcohol swab before injection. Then, 10 μL of each *Candida* suspension (10^6^ yeast cells) was inoculated directly to the last left pro-leg. An aliquot of 10 μL of 10.0 μM WMR was delivered behind the last proleg on the opposite side of the pathogen injection site either 2 h pre-infection (for prevention experiments) or 2 h post-infection (for treatment experiments). One group of untreated larvae served as a blank control group (intact larvae), one group received 10 μL of PBS solution per leg and one group was injected with 10 μL of WMR in one leg and 10 μL PBS in the other, to assess peptide toxicity. All groups of larvae were incubated at 35 °C in the dark [30,43,44]. Survival was recorded every day for 3 days. Larvae were considered dead if they gave no response to slight touch.

### 4.8. Time–Kill Kinetic Analysis

*C. albicans* and *C. parapsilosis* (10^6^ cells mL^−1^) were incubated with WMR at various concentrations (50.0, 100.0, 200.0 μM for the first and 10.0, 12.5, 25.0 μM for the second one) at 37 °C until 24 h. At time intervals of 6, 12 and 24 h, the cultures were spread on agar, incubated for 24/48 h at 37 °C, and the colony-forming units (CFU) were counted. All time–kill curve experiments were performed in triplicate [45].

### 4.9. Checkerboard Assays: Assessment of the In Vitro Synergy Activity of WMR Alone and in Combination with Fluconazole

The interaction of FLC and WMR against *C. albicans* and *C. parapsilosis* was assessed by the fractional inhibitory concentration index (FICI) model. The FICI was calculated for each agent by dividing the inhibition concentration of the antifungal combination by its MIC value. The calculation formula of the FICI model is as follows: FICI = (Ac/Aa) + (Bc/Ba), where Ac and Bc are the MIC values of tested agents in combination, while Aa and Ba correspond to these values for single-agent A and B treatments. A FICI of ≤ 0.5 means synergy; 0.5 < FICI ≤ 4 means no interaction; FICI > 4 means antagonism. Experiments were performed in triplicate [46].

### 4.10. Measurement of Intracellular ROS Levels and Mitochondrial Specific ROS Accumulation

Intracellular ROS levels were investigated using the fluorescent dye 2′,7′-dichlorofluorescein diacetate (DCFH-DA) (Molecular Probes, Eugene, OR, USA) while mitochondrial-specific ROS were measured by MitoSOX Red (Molecular Probes). *Candida* cells, with and without 10.0 μM WMR, after centrifugation at 13,000× *g* for 5 min, were treated with 10 mM H2DCFDA for 1 h, or 5 M MitoSOX Red (Molecular Probes), for 30 min at 37 °C. The fluorescent cells were measured with the FACS Verse microplate reader.

### 4.11. Cell Rescue Assay Using ROS Scavengers

For ROS quenching, N-acetyl cysteine (NAC) and glutathione were used (Sigma-Aldrich, St. Louis, MO, USA). Briefly, 10^6^ cells mL^−1^ cells were suspended in 12.5 mM sodium acetate and incubated at 37 °C with either 200 µM NAC or 32 µM glutathione for 30 min, respectively. Cells were harvested, washed with 12.5 mM sodium acetate, treated with or without different concentrations of WMR, (50.0 µM for *C. albicans* and 10.0 µM for *C. parapsilosis*) and incubated at 37 °C for 1 h. Cells were then plated on TSB agar plates and incubated overnight at 37 °C, and the number of CFUs from cells treated was counted and normalized to that of the untreated control. The results were reported as percentages of survival using the following formula: [(CFU of the sample treated with the agent)/(CFU of non-treated control) × 100] [47]. The data represent the mean ± standard deviation for three independent experiments.

### 4.12. Rh6G Efflux Assay

The cells were collected and washed three times with PBS and adjusted to 1 × 10^6^ cells mL^−1^. To fully deplete the energy of the cells, they were shaken for 1 h in the shaking incubator. Then, Rh6G was added at a final concentration of 10 mM and incubated at 37 °C for another 1 h and later transferred to an ice-water bath for 30 min to stop the uptake of Rh6G. WMR was then added alone or in combination with FLC and at time intervals of 0, 40, 80, 120, 160, and 200 min, the fluorescence intensity of intracellular Rh6G was measured using a microplate reader with excitation wavelength at 488 nm and emission wavelength at 530 nm. The experiment was repeated three times independently [29,48].

### 4.13. CLSM Analysis

The activity of WMR against biofilms of *C. parapsilosis* was evaluated by Confocal Laser Scanning Microscopy (CLSM). Biofilms were formed on NuncTM Lab-Tek^®^ 8-well Chamber Slides (n◦17744; Thermo Scientific, Ottawa, ON, Canada). Briefly, the wells of the chamber slide were filled with 300 μL of an overnight cultures of *C. parapsilosis* diluted at 1 × 10^6^ colony forming units (CFU) mL^−1^. The culture was incubated at 37 °C for 24 h in the absence and in the presence of WMR (10.0 μM) to assess its antibiofilm activity and its influence on cell viability. The biofilm cell viability was determined by the FilmTracer^TM^ LIVE/DEAD^®^ Biofilm Viability Kit (Molecular Probes, Invitrogen, Carlsbad, CA, USA), following the manufacturer’s instructions. After rinsing with filter-sterilized PBS, each well of the chamber slide was filled with 300 μL of working solution of fluorescent stains, containing SYTO^®^9 green-fluorescent nucleic acid stain (10 μM) and propidium iodide, the red-fluorescent nucleic acid stain (60 μM), and incubated for 20–30 min at room temperature, protected from light. All excess stain was removed by rinsing gently with filter-sterilized PBS. All microscopic observations and image acquisitions were performed with a confocal laser scanning microscope (LSM700-Zeiss, Jena, Germany) equipped with an Ar laser (488 nm), and a He-Ne laser (555 nm). Images were obtained using a 20x/0.8 objective. The excitation/emission maxima for these dyes are 480/500 nm for SYTO^®^9 and 490/635 nm for PI. Z-stacks were obtained by driving the microscope to a point just out of focus on both the top and bottom of the biofilms. Images were recorded as a series of .tif files with a file-depth of 16 bits. The COMSTAT software package [49] was used to determine biomasses (μm^3^ μm^−2^), average thicknesses (μm) and roughness coefficient (Ra*). For each condition, two independent biofilm samples were used.

### 4.14. Localization of FITC-Labeled Peptide in C. parapsilosis

Overnight cultures of *C. parapsilosis* were diluted to 0.2 OD_600_ in a solution of TSB 2% (*v*/*v*) in PBS, then the bacterial cells were treated with FITC-labeled WMR at a concentration of 10.0 µM at 37 °C for different incubation times (5 min, 2 h, 4 h, 6 h). Untreated cells served as a negative control. Then, the cells were stained with Calcofluor white stain (CFW), which binds to cellulose in cell walls. After 15 min incubation in the dark, the cells (5 μL) were spotted on a microscope slide and covered with a coverslip. Localization of the peptide was performed with a confocal laser scanning microscope (CLSM; LSM700-Zeiss, Jena, Germany) equipped with an Ar laser (488 nm), and a He-Ne laser (555 nm). Images were obtained using a 63×/0.8 objective The excitation/emission maxima for these dyes are 355/433 nm for CFW and 488/530 nm for FITC. Images were recorded as a series of .tif files with a file depth of 16 bits.

### 4.15. Statistical Analysis

All graphs were made with GraphPad Prism Software (version 8.02 for Windows, GraphPad Software, La Jolla, CA, USA, www.graphpad.com accessed on 20 December 2021). The results reported are the mean values and standard deviation (SD) obtained from three different experiments. For inhibition and eradication, levels of ROS and ROS scavengers were analyzed using one-way analysis of variance (ANOVA) followed by Holm–Sidak’s test. Time to kill of WMR and Rh6G efflux were analyzed using Student’s *t*-test. Survival curves were plotted using the Kaplan–Meier method and the differences between groups were analyzed using Dunnett’s multiple comparation test. Data with *p*-values < 0.05 were considered statistically significant.

## Figures and Tables

**Table 1:** Minimal inhibitory concentration (MIC) and minimal fungicide concentration (MFC) of WMR (µM) and fluconazole (µM) against *Candida* strains. FLC = fluconazole.

Strains	WMR (µM)		FLC (µM)	
	MIC	MFC	MIC	MFC
*C. albicans*	>50.0	>50.0	>163.0	>163.0
*C. auris*	>50.0	>50.0	163.0	>163.0
*C. glabrata*	>50.0	>50.0	>163.0	>163.0
*C. tropicalis*	25.0	50.0	81.5	>163.0
*C. parapsilosis*	25.0	25.0	16.3	32.6

## Data Availability

Not applicable.

## Supplementary Materials

The following supporting information can be downloaded at: https://www.mdpi.com/article/10.3390/ijms23042151/s1.
