# Supplementary material for: WMR Peptide as Antifungal and Antibiofilm against Albicans and Non-Albicans Candida Species: Shreds of Evidence on the Mechanism of Action
Source: Int J Mol Sci. 2022 Feb 15;23(4):2151. doi: 10.3390/ijms23042151 (PMC8879636; doi:10.3390/ijms23042151)
Supplement: Supplementary file 1 [file ijms-23-02151-s001.zip › ijms-1589683-supplementary.pdf]

Table. S1 Name, acronym, sequence and references of used primer for *RT-qPCR.*

Gene names	*Acronym*	Primer name	Sequence (5'→3')	Reference
Agglutinin like-sequence 3	*ALS3*	C.albicans_ALS3_F<br>C.albicans_ALS3_R	CTAATGCTGCTACGTATAATT<br>CCTGAAATTGACATGTAGCA	[1]
Genes encoding efflux pumps	*CDR1*	C.albicans_CDR1_F<br>C.albicans_CDR1_R	CAGCCACTGAAACACCAACT<br>CAGAAGTAACAACAACAACACCAG	[2]
Ergosterol biosynthesis enzyme	*ERG11*	C.albicans_ERG11_F<br>C.albicans_ERG11_R	ATTGTTGAAACTGTCATTG<br>CCCCTAATAATATACTGATCTG	[2]
High-osmolarity glycerol1	*HOG1*	C.albicans_HOG1_F<br>C.albicans_HOG1_R	GACTTGTGGTCTGTGGGTTG<br>ACATCAGCAGGAGGTGAGC	[3]
Actin	*actin*	C.albicans_actin_F<br>C.albicans_actin_R	AGCCCAATCCAAAAGAGGTATT<br>GCTTCGGTCAACAAAACTGG	[1]
Agglutinin like-sequence 3	*ALS3*	C.glabrata_ALS3_F<br>C.glabrata_ALS3_R	CTGGACCACCAGGAAACACT<br>GGTGGAGCGGTGACAGTAGT	[4]
Genes encoding efflux pumps	*CDR1*	C.glabrata_CDR1_F<br>C.glabrata_CDR1_R	TAGCACATCAACTACACGAACGT<br>AGAGTGAACATTAAGGATGCCATG	[5]
Ergosterol biosynthesis enzyme	*ERG11*	C.glabrata_ERG11_F<br>C.glabrata_ERG11_R	ATTGGTGTCTTGATGGGTGGTC<br>TCTTCTTGGACATCTGGTCTTTCA	[5]
High-osmolarity glycerol1	*HOG1*	C.glabrata_HOG1_F<br>C.glabrata_HOG1_R	GACTTGTGGTCTGTGGGTTG<br>ACATCAGCAGGAGGTGAGC	[3]
Actin	*actin*	C.glabrata_actin_F<br>C.glabrata_actin_R	TTGCCACACGCTATTTTGAG<br>ACCATCTGGCAATTCGTAGG	[1]
Agglutinin like-sequence 5	*ALS5*	C.auris_ALS5_F<br>C.auris_ALS5_R	CCTTCTGGATCGGACACAGT<br>AGTTGTGGTGGAGGAACCAG	[6]
Genes encoding efflux pumps	*CDR1*	C.auris_CDR1_F<br>C.auris_CDR1_R	GAAATCTTGCACTTCCAGCCC<br>CATCAAGCAAGTAGCCACCG	[6]
Ergosterol biosynthesis enzyme	*ERG11*	C.auris_ERG11_F<br>C.auris_ERG11_R	GTGCCCATCGTCTACAACCT<br>TCTCCCACTCGATTTCTGCT	[6]
High-osmolarity glycerol1	*HOG1*	C.auris_HOG1_F<br>C.auris_HOG1_R	GACTTGTGGTCTGTGGGTTG<br>ACATCAGCAGGAGGTGAGC	[3]
Actin	*actin*	C.auris_actin_F<br>C.auris_actin_R	GAAGGAGATCACTGCTTTAGCC<br>GAGCCACCAATCCACACAG	[6]
Agglutinin like-sequence 3	*ALS3*	C.tropicalis_ALS3_F<br>C.tropicalis_ALS3_R	AGGTGCTGTAGTTGTTCTT<br>AGCAGTCGGGTTGAAAGG	[7]
Genes encoding efflux pumps	*CDR1*	C.tropicalis_CDR1_F<br>C.tropicalis_CDR1_R	AGACAATCAGAGCACACT<br>AACCGAAGACAATATCAATCC	[7]
Ergosterol biosynthesis enzyme	*ERG11*	C.tropicalis_ERG11_F<br>C.tropicalis_ERG11_R	TTGATTGATTCCTTGTTGGTTA<br>CATCTTGTAATTGTGGTTGTTC	[8]
High-osmolarity glycerol1	*HOG1*	C.tropicalis_HOG1_F<br>C.tropicalis_HOG1_R	GACTTGTGGTCTGTGGGTTG<br>ACATCAGCAGGAGGTGAGC	[3]
Actin	*actin*	C.tropicalis_actin_F<br>C.tropicalis_actin_R	GGCTGGTAGAGACTTGACCAACCATTTG<br>GGAGTTGAAAGTGGTTTGGTCAATAC	[9]
Agglutinin like-sequence 3	*ALS3*	C.parapsilosis_ALS3_F<br>C.parapsilosis_ALS3_R	AAGTCGAGACCCACCCATTG<br>TTGTGTCCCTTTGCACTGCC	[10]
Genes encoding efflux pumps	*CDR1*	C.parapsilosis_CDR1_F<br>C.parapsilosis_CDR1_R	GCTGTTGATCAAAGGGGTGT<br>ATCCAAAATCCAGGCAACTG	[11]
Ergosterol biosynthesis enzyme	*ERG11*	C.parapsilosis_ERG11_F<br>C.parapsilosis_ERG11_R	TGTTGCATTTGGCTGAGAAG<br>TCTGAGGGTTTCCTTGATGG	[11]
High-osmolarity glycerol1	*HOG1*	C.parapsilosis_HOG1_F<br>C.parapsilosis_HOG1_R	GACTTGTGGTCTGTGGGTTG<br>ACATCAGCAGGAGGTGAGC	[3]
Actin	*actin*	C.parapsilosis_actin_F<br>C.parapsilosis_actin_R	ACGGTATTGTTTCCAACTGGGACG<br>TGGAGCTTCGGTCAACAAAACTGG	[11]

References

1. Alves, C.T., et al., *Candida albicans promotes invasion and colonisation of Candida glabrata in a reconstituted human vaginal epithelium.* J Infect, 2014. **69**(4): p. 396-407.

2. Ivanov, M., et al., *Flavones, Flavonols, and Glycosylated Derivatives-Impact on Candida albicans Growth and Virulence, Expression of CDR1 and ERG11, Cytotoxicity.* Pharmaceuticals (Basel), 2020. **14**(1).
3. Wang, L., et al., *SPT20 Regulates the Hog1-MAPK Pathway and Is Involved in Candida albicans Response to Hyperosmotic Stress.* Front Microbiol, 2020. **11**: p. 213.
4. Morse, D.J., et al., *Denture-associated biofilm infection in three-dimensional oral mucosal tissue models.* J Med Microbiol, 2018. **67**(3): p. 364-375.
5. Sakagami, T., et al., *Antifungal susceptibility trend and analysis of resistance mechanism for Candida species isolated from bloodstream at a Japanese university hospital.* J Infect Chemother, 2019. **25**(1): p. 34-40.
6. Bhattacharya, S., et al., *Gene Duplication Associated with Increased Fluconazole Tolerance in Candida auris cells of Advanced Generational Age.* Sci Rep, 2019. **9**(1): p. 5052.
7. Yu, S., et al., *Distinct Expression Levels of ALS, LIP, and SAP Genes in Candida tropicalis with Diverse Virulent Activities.* Front Microbiol, 2016. **7**: p. 1175.
8. Fan, X., et al., *Molecular mechanisms of azole resistance in Candida tropicalis isolates causing invasive candidiasis in China.* Clin Microbiol Infect, 2019. **25**(7): p. 885-891.
9. Losberger, C. and J.F. Ernst, *Sequence of the Candida albicans gene encoding actin.* Nucleic Acids Res, 1989. **17**(22): p. 9488.
10. Oh, S.H., et al., *Agglutinin-Like Sequence (ALS) Genes in the Candida parapsilosis Species Complex: Blurring the Boundaries Between Gene Families That Encode Cell-Wall Proteins.* Front Microbiol, 2019. **10**: p. 781.
11. Neji, S., et al., *Virulence factors, antifungal susceptibility and molecular mechanisms of azole resistance among Candida parapsilosis complex isolates recovered from clinical specimens.* J Biomed Sci, 2017. **24**(1): p. 67.

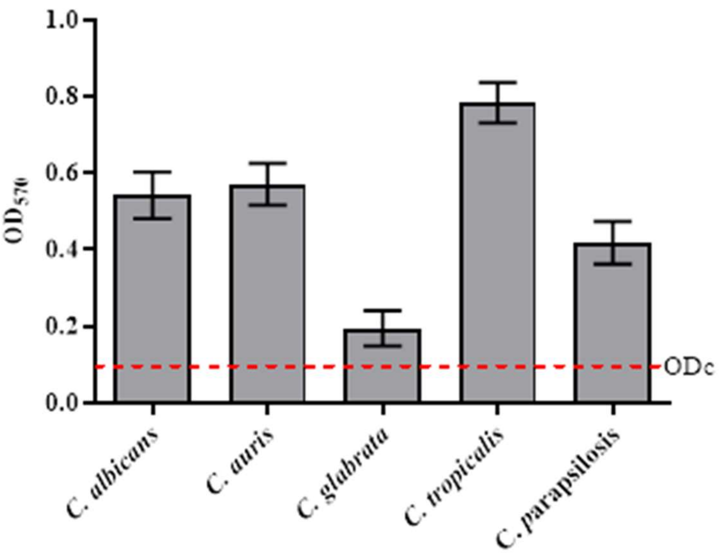

Figure.S1. Biofilm formation capacity of five species microorganisms using crystal violet staining method. OD cut = mean of negative control with 3 times addition of SD.

		WMR				
		C.glabrata	*C.auris*	*C.parapsilosis*	*C.tropicalis*	*C.albicans*
Stress	*HOG1*	-4.38	-3.54	4.93	-10.93	-0.05
Structure formation	*ERG11*	-1.88	9.52	2.14	-6.19	0.32
Biofilms formation	*ALS3/5*	-2.52	-3.62	1.76	-7.42	-2.76
Efflux pumps	*CDR1*	-0.33	-10.17	-4.41	-8.47	-1.61

Table.S2 Data of expression levels in fungal biofilms, exposed to WMR at 10 μM, were reported as a fold difference (in green and red down- and up- expressed genes) from control (represented by biofilm untreated). Fold differences greater than ± 1.5 were considered significant.

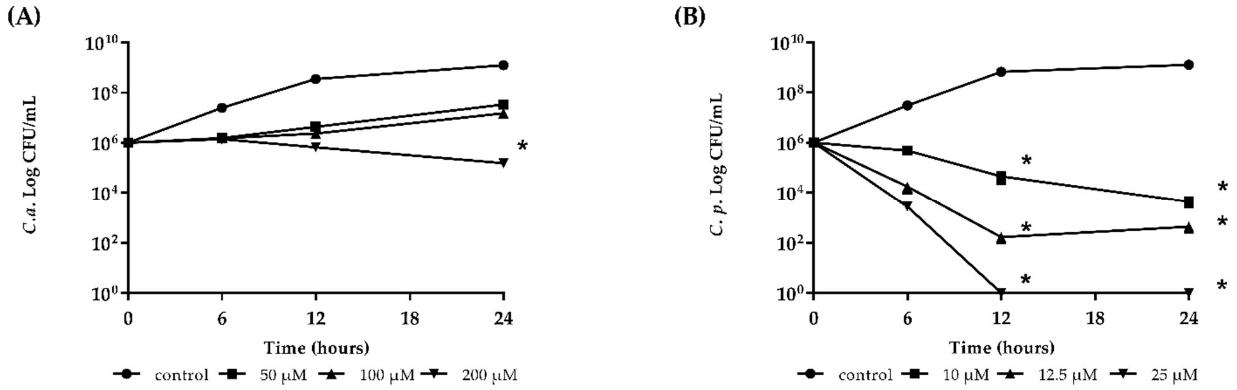

Figure. S2. Time-kill kinetics of WMR against *C. albicans* and *C.parapsilosis*. (A) Growth curves generated using *C. albicans* cells treated with WMR (50; 100; 200 μM) (B) Growth curves generated using *C. parapsilosis* cells treated WMR (10; 12.5; 25 μM). (n=3 ± SD); * $p < 0.05$ (two-way ANOVA followed by Tukey's post hoc test).

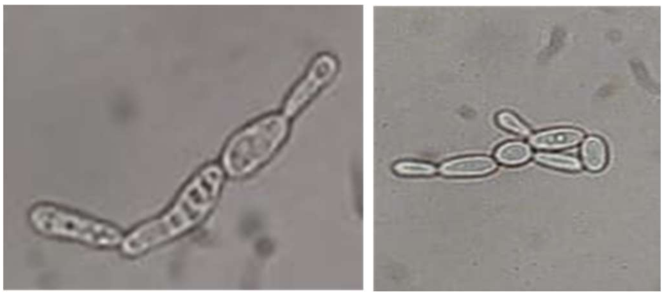

Figure. S3. Analysis of the WMR effect on the *C. parapsilosis* cells. Microscopy images of *C. parapsilosis* cells formed in the presence (10μM) and absence (NT) of WMR.
